# Structural priors represented by discrete cosine transform improve EIT functional imaging

**DOI:** 10.1371/journal.pone.0285619

**Published:** 2023-05-11

**Authors:** Rongqing Chen, Sabine Krueger-Ziolek, András Lovas, Balázs Benyó, Stefan J. Rupitsch, Knut Moeller

**Affiliations:** 1 Institute of Technical Medicine (ITeM), Furtwangen University, Villingen-Schwenningen, Germany; 2 Faculty of Engineering, University of Freiburg, Freiburg, Germany; 3 Department of Anaesthesiology and Intensive Therapy, Kiskunhalas Semmelweis Hospital, Kiskunhalas, Hungary; 4 Department of Control Engineering and Information Technology, Faculty of Electrical Engineering and Informatics, Budapest University of Technology and Economics, Budapest, Hungary; Khalifa University of Science and Technology, UNITED ARAB EMIRATES

## Abstract

Structural prior information can improve electrical impedance tomography (EIT) reconstruction. In this contribution, we introduce a discrete cosine transformation-based (DCT-based) EIT reconstruction algorithm to demonstrate a way to incorporate the structural prior with the EIT reconstruction process. Structural prior information is obtained from other available imaging methods, e.g., thorax-CT. The DCT-based approach creates a functional EIT image of regional lung ventilation while preserving the introduced structural information. This leads to an easier interpretation in clinical settings while maintaining the advantages of EIT in terms of bedside monitoring during mechanical ventilation. Structural priors introduced in the DCT-based approach are of two categories in terms of different levels of information included: a contour prior only differentiates lung and non-lung region, while a detail prior includes information, such as atelectasis, within the lung area. To demonstrate the increased interpretability of the EIT image through structural prior in the DCT-based approach, the DCT-based reconstructions were compared with reconstructions from a widely applied one-step Gauss-Newton solver with background prior and from the advanced GREIT algorithm. The comparisons were conducted both on simulation data and retrospective patient data. In the simulation, we used two sets of forward models to simulate different lung conditions. A contour prior and a detail prior were derived from simulation ground truth. With these two structural priors, the reconstructions from the DCT-based approach were compared with the reconstructions from both the one-step Gauss-Newton solver and the GREIT. The difference between the reconstructions and the simulation ground truth is calculated by the *ℓ*_2_-norm image difference. In retrospective patient data analysis, datasets from six lung disease patients were included. For each patient, a detail prior was derived from the patient’s CT, respectively. The detail prior was used for the reconstructions using the DCT-based approach, which was compared with the reconstructions from the GREIT. The reconstructions from the DCT-based approach are more comprehensive and interpretable in terms of preserving the structure specified by the priors, both in simulation and retrospective patient data analysis. In simulation analysis, the *ℓ*_2_-norm image difference of the DCT-based approach with a contour prior decreased on average by 34% from GREIT and 49% from the Gauss-Newton solver with background prior; for reconstructions of the DCT-based approach with detail prior, on average the *ℓ*_2_-norm image difference is 53% less than GREIT and 63% less than the reconstruction with background prior. In retrospective patient data analysis, the reconstructions from both the DCT-based approach and GREIT can indicate the current patient status, but the DCT-based approach yields more interpretable results. However, it is worth noting that the preserved structure in the DCT-based approach is derived from another imaging method, not from the EIT measurement. If the structural prior is outdated or wrong, the result might be misleadingly interpreted, which induces false clinical conclusions. Further research in terms of evaluating the validity of the structural prior and detecting the outdated prior is necessary.

## Introduction

Electrical Impedance Tomography (EIT) is a functional radiation-free imaging technique, which measures regional lung ventilation and aeration distribution. Electrodes that are placed equidistantly between the 4th and the 5th intercostal space around the chest, are used to stimulate with a weak alternating electric current and to measure induced changes in electrical potentials at the skin surface of the chest during breathing. A typical EIT scan delivers 20-50 images per second [1–4]. It thus shows a higher time resolution but lower spatial resolution (usually 2-3cm) than other common imaging methods such as computed tomography (CT) and magnetic resonance imaging (MRI) [5–7]. In addition, the cost of EIT is considerably lower compared to other functional imaging methods like single photon emission computed tomography (SPECT) or positron emission tomography (PET) [8–10]. As a modern diagnosis and monitoring tool, EIT has shown the potential to guide mechanical ventilation [11–13]. It helps to identify overdistention of the anterior part or intratidal recruitment in the dependent part, which is evaluated by clinicians to optimize positive end-expiratory pressure (PEEP) [14, 15]. Eventually, this will lower the risk of ventilator induced lung injury (VILI) [14, 16, 17]. EIT is also applicable in adult patients with ARDS [18, 19], chronical obstructive pulmonary disease (COPD) [20, 21], or cystic fibrosis [22]. Recently, the optimal settings of mechanical ventilation therapy has attracted much attention due to the increasing cases of COVID-19 infections of which 5-15% require intensive care surveillance and mechanical ventilation support [23, 24].

In practice, though, EIT images are difficult to interpret and results may depend on the reconstruction method employed [25–27]. There have been several advancements in the development of EIT algorithms, each with its own unique advantages. Many of these methods have been specifically designed for certain purposes, such as compensating for motion artifacts [28], or faulty electrodes [29], 3D imaging [30], noise reduction through temporal correlation [31], and utilizing Kalman filter approaches for tracking fast changes in conductivity distribution [32]. The D-bar algorithm has been developed for absolute EIT imaging [33]. The GREIT is based on expert-defined figures of merit [34]. The orthonormal eigenimages from CT data are used as training sets [35]. Approaches with the level set method [36], or Total Variation regularization [37], are used to prevent blurring of reconstructed images. With the constantly increasing computational power and parallel computing capabilities, there is a rise in learning-based inverse solvers for EIT, such as the EIT image reconstruction based on structure-aware sparse Bayesian learning [38, 39]. The algorithms utilizing Deep Neural Networks, e.g., deep D-bar algorithm [40], have been reported to enable real-time reconstruction of absolute EIT images.

Despite these advancements, low spatial resolution combined with blurred anatomical alignment and reconstruction-induced artifacts still hinder the interpretation of patient status in clinical settings. Incorporating structural priors, such as a lung contour, into the EIT image reconstruction process can improve anatomic orientation and benefit clinicians [25]. Structural priors can be included in the reconstruction in different ways, for example, it can be included as uniformed weighting [34], as modified inverse FEM model [41], or in form of different kernel functions [42] or altogether.

Previously, a novel EIT reconstruction algorithm was proposed using the discrete cosine transformation (DCT) and structural priors obtained from CT data to improve the interpretability of EIT images [43]. It creates a patient specific anatomically oriented EIT reconstruction, which offers a natural way to preserve the original conductivity distribution, while improving image quality and readability. In addition, this algorithm provides a scalable dimensionality of the EIT reconstruction problem. By reducing the degree of freedom of the Jacobian matrix, this algorithm improves the real-time performance of EIT [43].

However, it only has a simple inclusion of a lung contour from CT as a structural prior in the reconstruction process. Additional information from CT, e.g., atelectasis area, which can also function as structural priors, are missing. As a lung contour only differentiates lung region and non-lung region, pathological details can be introduced as weights to enhance or to attenuate the impedance changes during the reconstruction process. The effect of these two different types of structural priors, which are called contour prior and detail prior, is not yet demonstrated on the reconstruction of the DCT-based approach. In addition, the DCT-based approach was not compared to other existing robust algorithms for reconstruction. Graz consensus Reconstruction algorithm for EIT (GREIT), for example, is an advanced and commonly used algorithm [27, 30, 44]. The one-step Gauss-Newton method also represents a broad group of structurally similar algorithms for image reconstruction, which have been widely applied in EIT [34, 45]. Thus, it is necessary to compare the reconstruction of the DCT-based approach with the reconstruction from both GREIT algorithm and one-step Gauss-Newton method. The objective of this work is to demonstrate the increased interpretability of the EIT images through structural priors integrated into the DCT-based reconstruction approach; and to evaluate the effect of different structural prior knowledge, which in the following is called DCT priors, inserted into the EIT reconstruction process. For the evaluation, we used simulations and retrospective patient data.

## Methods

### EIT image reconstruction

The reconstruction of an EIT image is ill-posed, which means there is no unique solution, therefore an optimization with regularization is needed to enforce unique estimates of the conductivity distribution *σ* from the boundary voltages **v** [46, 47]. In addition, the variation of the conductivity distribution **x** = *σ* − *σ*^*baseline*^ and the induced changes of the boundary voltages **y** = **v** − **v**^*baseline*^ are nonlinearly related [48]. The actual conductivity distribution can be inhomogeneous and nonlinear, thus, a finite element model (FEM) is commonly used to discretize the domain into piecewise constant regions [49].

The EIT inverse problem is ill-posed, i.e., regularization is required to constrain the solution space. It can be expressed in terms of a non-linear optimization problem:

$$\begin{array}{c} \hat{\text{x}}=\underset{\text{x}}{\text{argmin}}\{\|F\left(\text{x}\right)-y{\|}_{2}^{2}+\lambda^{2}\|\text{Rx}{\|}_{2}^{2}\} \end{array}$$ (1)

where $\hat{\text{x}}$ represents the reconstructed conductivity change, *F*(**x**) is the nonlinear model that maps the conductivity change in a model to the measured boundary voltage variation, **y** represents the measured boundary voltage changes, λ is a hyperparameter to control the influence of regularization on obtained solution [50]. If only small internal changes of the conductivity are assumed, a linear mapping *F*(**x**) ≈ **Jx** can be estimated:

$$\begin{array}{c} \text{y}\approx \text{Jx}+\text{n} \end{array}$$ (2)

where **n** represents an additive noise matrix superimposed on the boundary measurements. The matrix **J** is a Jacobian matrix, of which *J*_*i*,*j*_ maps a conductivity change at the FEM element *j* to an induced voltage change in the boundary position *i* at a baseline point:

$$\begin{array}{c} J_{i,j}=\frac{\partial y_{i}}{\partial x_{j}}{|}_{\sigma^{baseline}} \end{array}$$ (3)

With the previous assumption that the conductivity change is small, the Eq 1 is linearized:

$$\begin{array}{c} \hat{\text{x}}=\underset{\text{x}}{\text{argmin}}\{\|\text{Jx}-\text{y}{\|}_{2}^{2}+\lambda^{2}\|\text{Rx}{\|}_{2}^{2}\} \end{array}$$ (4)

where **R** is a regularization penalty to reconstruct the impedance distribution. **R** can be regarded as a traditional way to introduce a penalty to the ill-posed inverse problem to form a more well-posed problem. **R** can be selected from various choices, for example from Tikhonov prior [51], NOSER prior [52], or Laplace prior [53]. In this work, the Tikhonov prior **R** = **I** was chosen. The hyperparameter λ is optimized to reach a noise figure of 0.5 (*NF* = 0.5) as recommended [34]. The conductivity distribution estimation of the $\hat{\text{x}}$ can then be calculated in a closed form:

$$\begin{array}{c} \hat{\text{x}}={\left({\text{J}}^{T}\text{J}+\lambda^{2}{\text{R}}^{T}\text{R}\right)}^{-1}{\text{J}}^{T}\text{y}=\text{By} \end{array}$$ (5)

**B** is the reconstruction matrix which calculates the impedance distribution variation from the measured boundary voltages.

### Prior information in EIT

**Fig 1:**
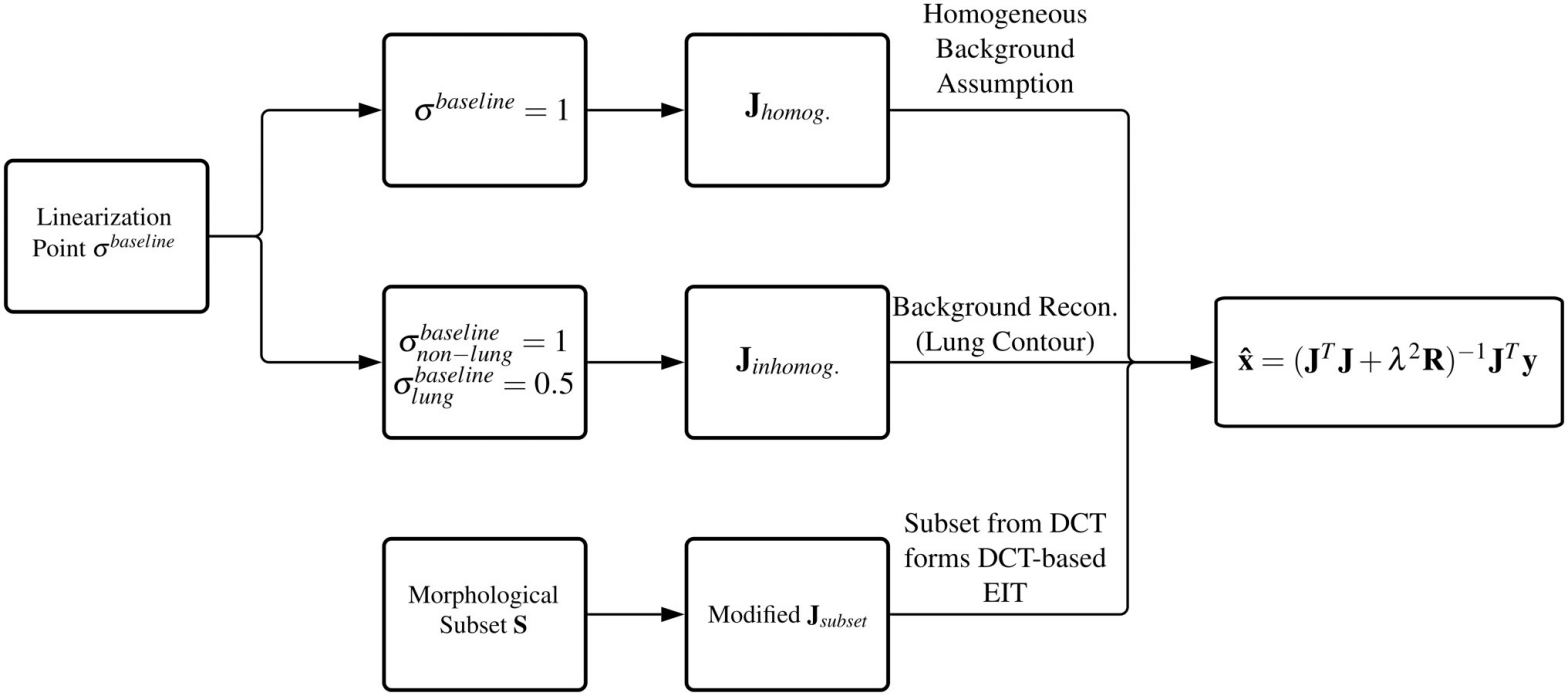
Calculation of the Jacobian matrix from different assumptions of the linearization point and the modification of the Jacobian matrix by a subset.

Apart from the introduction of a regularization penalty **R**, other options exist to include priors into EIT reconstruction, for example, anatomical structure can be introduced as a prior via the Jacobian matrix **J**. The calculation of Jacobian matrix **J** depends on the *σ*^*baseline*^ as a linearization point. Since the initial conductivity distribution is not known, it is common to assume a homogeneous conductivity distribution of *σ*^*baseline*^ = 1, which may cause some reconstruction errors [54, 55]. To avoid errors caused by the assumption of an invalid homogeneous prior, Grychtol et al. suggested that the setting of the *σ*^*baseline*^ could consider the conductivity distribution from the CT or MRI data of the specific patient to improve the image quality [55]. This method usually leads to clusters of FEM elements, which are topological neighbours and belong to the same grey values in the respective CT or MRI morphological image. For reconstruction, cluster elements are assigned the same property values. In our research, the linearization point *σ*^*baseline*^ was set as $\sigma_{lung}^{baseline}=0.5$ and $\sigma_{non-lung}^{baseline}=1$ to form a better prior separating between lung and background in the calculation of the Jacobian matrix **J**. This method was used to exemplify a typical approach to implement a lung contour prior into the EIT reconstruction process. Fig 1 illustrates the different assumptions of the linearization point *σ*^*baseline*^ to calculate the final Jacobian matrix **J**.

In addition to FEM element clustering, priors can be integrated into a subset of basic functions, e.g., the basis constraint method [50]. Thus, the matrix **J** is modified to the reconstruction matrix ${\text{J}}_{subset}^{n_{meas}\times n_{subset}}={\text{J}}^{n_{meas}\times n_{elems}}S^{n_{elems}\times n_{subset}}$ (Fig 1). *n*_*meas*_ is the number of boundary measurements in EIT reconstruction, *n*_*elems*_ is the number of elements in the FEM model, and *n*_*subset*_ is the number of basic functions in the modified subsets, or the dimensionality of the subsets. If the dimensionality of the subsets is small compared to *n*_*elems*_ (*n*_*subset*_ ≪ *n*_*elems*_), the degrees of freedom of the inverse problem will be reduced to the number of the subset groups, and thus ill-posed characteristic of the optimization problem can be controlled. Suitable kernel functions can be chosen for such subsets. An efficient reverse transform must exist for the kernel functions to recover an image. The original Jacobian matrix **J** maps conductivity changes within the FEM elements to induced voltage changes at the boundary. The subset modified Jacobian matrix **J**_*subset*_ maps the changes of the subsets within grouped FEM elements to the induced voltage changes and, thus, integrates kernel functions into standard reconstruction. A final EIT reconstruction is restored from the estimated change of the subsets. Any image compression algorithm is principally an appropriate candidate method to generate subsets, e.g., discrete cosine transformation (DCT) or wavelet transformation or others.

### DCT based EIT reconstruction

In our research, the basic cosine functions from DCT are used to generate the modifying subsets. The solution of the inverse problem with the modified Jacobian matrix **J**_*DCT*_ is represented by the change of the DCT coefficients. DCT is a prominent and widely used method in image processing, e.g., in JPEG image compression [56]. The concept of DCT is to represent the image with a sum of cosine functions of varying frequencies, *n*_*xDCT*_ in x-direction and *n*_*yDCT*_ in y-direction respectively. Those add up to the total DCT order *n*_*DCT*_ = *n*_*xDCT*_ ⋅ *n*_*yDCT*_. For a two-dimensional image **A** with *M* rows and *N* columns, the DCT process is described as:

$$\begin{array}{c} {\text{V}}_{p,q}=\alpha_{p}\alpha_{q}\sum_{m=0}^{M-1}\sum_{n=0}^{N-1}A_{m,n}\cdot \text{cos}\frac{(2m+1)p\pi }{2M}\cdot \text{cos}\frac{(2n+1)q\pi }{2N} \end{array}$$ (6)

where

$$\alpha_{p}=\{\begin{array}{cc} \frac{1}{\sqrt{M}} & p=0\\ \sqrt{\frac{2}{M}} & 1\leq p\leq M-1 \end{array}$$

and

$$\alpha_{q}=\{\begin{array}{cc} \frac{1}{\sqrt{N}} & q=0\\ \sqrt{\frac{2}{N}} & 1\leq q\leq N-1 \end{array}$$

The matrix **V** consists of the DCT coefficients, of which a subset can roughly recover a compressed image $\tilde{\text{A}}$ of **A**:

$$\begin{array}{c} {\tilde{A}}_{m,n}=\sum_{p=0}^{M-1}\sum_{q=0}^{N-1}\alpha_{p}\alpha_{q}{\tilde{V}}_{m,n}\cdot \text{cos}\frac{(2m+1)p\pi }{2M}\cdot \text{cos}\frac{(2n+1)q\pi }{2N} \end{array}$$ (7)

where $\tilde{\text{V}}$ is a sparse matrix of the same size as **V** but only having several non-zero elements from **V**.

The sparse matrix $\tilde{\text{V}}$ is of DCT order *n*_*DCT*_. In our contribution, the DCT order was set to *n*_*DCT*_ = 225, which is composed of 15 frequencies in both *x*-direction and *y*-direction (*p*, *q* ∈ (0, 1, …, 14)). *p* represents DCT frequencies in *x*-direction, and *q* in *y*-direction. The choice of DCT order *n*_*DCT*_ depends on the distribution of frequencies derived from image **A**. Empirical tests on our data revealed that information beyond 15 frequencies in both *x*-direction and *y*-direction is negligible in our application.

The different frequencies combinations *p*, *q* of the basic cosine functions from sparse $\tilde{\text{V}}$ can be written as a matrix **D**(*p*, *q*):

$$\begin{array}{c} \text{D}\left(p,q\right)=\alpha_{p}\alpha_{q}\cdot \text{cos}\frac{(2m+1)p\pi }{2M}\cdot \text{cos}\frac{(2n+1)q\pi }{2N}\;\;\;\;\;\left(p,q\in (0,1,\ldots ,14)\right) \end{array}$$ (8)

It is worth noting that **D**(*p*, *q*)_*m*,*n*_ does not include the image information, i.e., pixel value *A*_*m*,*n*_, but just the basic cosine functions. With the aim to restrict reconstruction to the lung area, $\text{C}\in R^{M\times N\times n_{DCT}}$ was generated to include the structural prior:

$$\begin{array}{c} C{\left(p,q\right)}_{m,n}=P_{m,n}\cdot D{\left(p,q\right)}_{m,n} \end{array}$$ (9)

of which matrix **P** is the structural prior derived from a morphological image, such as a CT image **A**^*CT*^. For this presentation, we classify structural priors in the DCT-based approach into two groups. A structural prior **P** can be binary and limits the reconstruction to the lung area, but does not include further anatomical structures or pathophysiological changes within the lung area. This prior is called a contour prior (cf. Fig 3) and denoted as **P**^*cp*^:

$$P_{m,n}^{cp}=\{\begin{array}{cc} 1 & elements\;within\;lung\\ 0 & elements\;outside\;lung \end{array}$$

A structural prior **P** may include anatomical or pathophysiological details derived from morphological images, e.g., using the atelectasis area shown in CT to constrain the impedance change within the same area in the EIT reconstruction (cf. Fig 3). This type of structural prior is called detail prior **P**^*dp*^, whose element values vary between 0 and 1 ($0\leq P_{m,n}^{dp}\leq 1$). In our work, we used Hounsfield units of CT image **A**^*CT*^, but any other reasonable assignment is possible too. Pure air is -1,000 Hounsfield units, normal lung tissue is -700 Hounsfield units (70% air, 30% tissue), while Hounsfield units for atelectasis, edema, and infiltrates are close to 0 [57]. Thus, the detail prior derived from **A**^*CT*^ is calculated as:

$$P_{m,n}^{dp}=\{\begin{array}{cc} \frac{A_{m,n}^{CT}}{-1000} & elements\;within\;lung\\ 0 & elements\;outside\;lung \end{array}$$

These different priors will lead to different levels of constraints within the reconstructions. In general, it is possible to include any appropriate information that can be obtained, e.g., from an image source mapped to a matrix of appropriate dimension (*M* × *N*) and then introduced into EIT reconstruction. The elements from the most ventilated area are set to 1, and the elements from the not ventilated area are set to 0. Other elements within lung region are scaled between 0 to 1. Structural priors from different morphological images may introduce same structural constrains to the EIT reconstruction (like a lung contour). The choice of modality is based on the availability and applicability.

**Fig 2:**
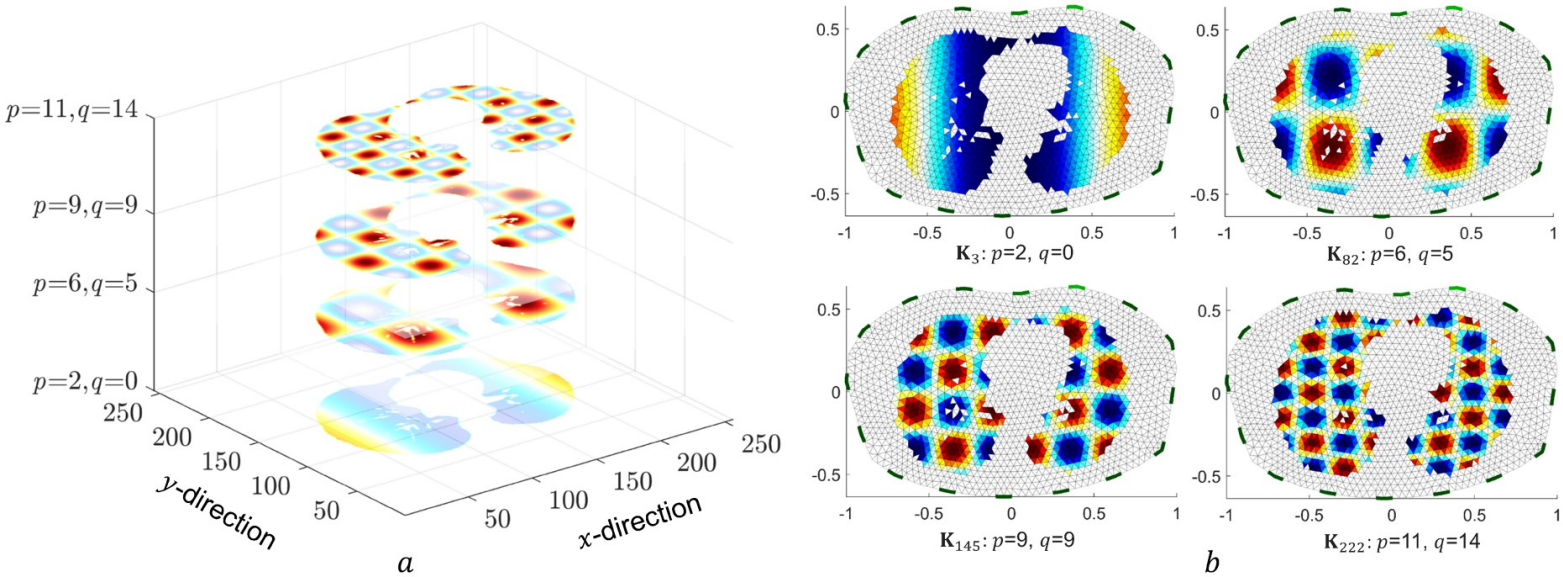
Left: An example of **C** with four different frequencies combinations *p*, *q*. Right: Visualization of the corresponding column of **K** with the FEM model: upper (left to right): **K**_3_, **K**_82_; lower (left to right): **K**_145_, **K**_222_.

An example of matrix **C** is illustrated in Fig 2a. Four different frequencies combinations are depicted. Please note that in Fig 2a, the projection **P** onto the lung area is already included.

From **C**(*p*, *q*), each column **K**_*j*_ of **K** is created as follows:

$$\begin{array}{c} {\text{K}}_{j}=T\left(\text{C}\left(p,q\right)\right) \end{array}$$ (10)

where *T* is a map to assign every pixel in **C**(*p*, *q*) to the element in the FEM model, which covers the pixel. *j* is the column index of the matrix **K**. It is calculated as $j\left(p,q\right)=q\cdot \sqrt{N_{DCT}}+p+1$, e.g., *p* = 2, *q* = 0 i.e. **K**_3_ = *T*(*C*(2, 0)). Each column **K**_*j*_ can be derived by the linear mapping *T* that assigns frequency dependent matrix **C**(*p*, *q*) (*p*, *q* ∈ (0, 1, …, 14)) to the FEM elements. Four different columns of matrix **K**, namely **K**_3_, **K**_82_, **K**_145_ and **K**_222_, are illustrated in Fig 2b. The number of pixels in the lung area of the matrix **C**(*p*, *q*) can be larger than the number of FEM elements. Thus, it is possible that not all elements of **C**(*p*, *q*) are used, but the columns of **K** are still linearly independent. In other words, **K** is a valid basis of the selected low frequency subspace of the DCT representation, containing only masked patterns of basis cosine functions. Multiplying the Jacobian to obtain **J**_*DCT*_ with **K** establishes the mapping from the DCT parameters ${\hat{\text{x}}}_{DCT}$ to the boundary measurements **y**.

After calculation of the subset matrix **K**, the implementation of the reconstruction matrix **B** is based on the subset modified reduced Jacobian **J**_*subset*_ = **J** ⋅ **S**. In the DCT-based reconstruction, the modifying subset is the DCT generated subset matrix ${\text{K}}^{n_{elems}\times n_{DCT}}$. Multiplying the Jacobian matrix ${\text{J}}^{n_{meas}\times n_{elems}}$ by the matrix ${\text{K}}^{n_{elems}\times n_{DCT}}$ allows the infusion of the structural prior into the EIT reconstruction process. As a result, the final Jacobian matrix **J**_*DCT*_ contains only *n*_*meas*_ × *n*_*DCT*_ elements, which are significantly fewer than the number of elements in a FEM used in the classical EIT reconstruction [43]. This new Jacobian matrix **J**_*DCT*_ can be used like the former Jacobian in the inverse process:

$$\begin{array}{c} {\hat{\text{x}}}_{DCT}={\left({\text{J}}_{DCT}^{T}{\text{J}}_{DCT}+\lambda^{2}{\text{R}}^{T}\text{R}\right)}^{-1}{\text{J}}_{DCT}^{T}\text{y}={\text{B}}_{DCT}\text{y} \end{array}$$ (11)

where the reconstruction matrix **B**_*DCT*_ maps the voltage variations to the change of DCT coefficients ${\hat{\text{x}}}_{\text{DCT}}\in R^{n_{DCT}}$. Throughout this contribution, the Tikhonov prior **R** is used for regularization. Thus, in Eq 11, **R** = **I** with the hyperparameter λ being optimized to reach a noise figure of 0.5.

**Fig 3:**
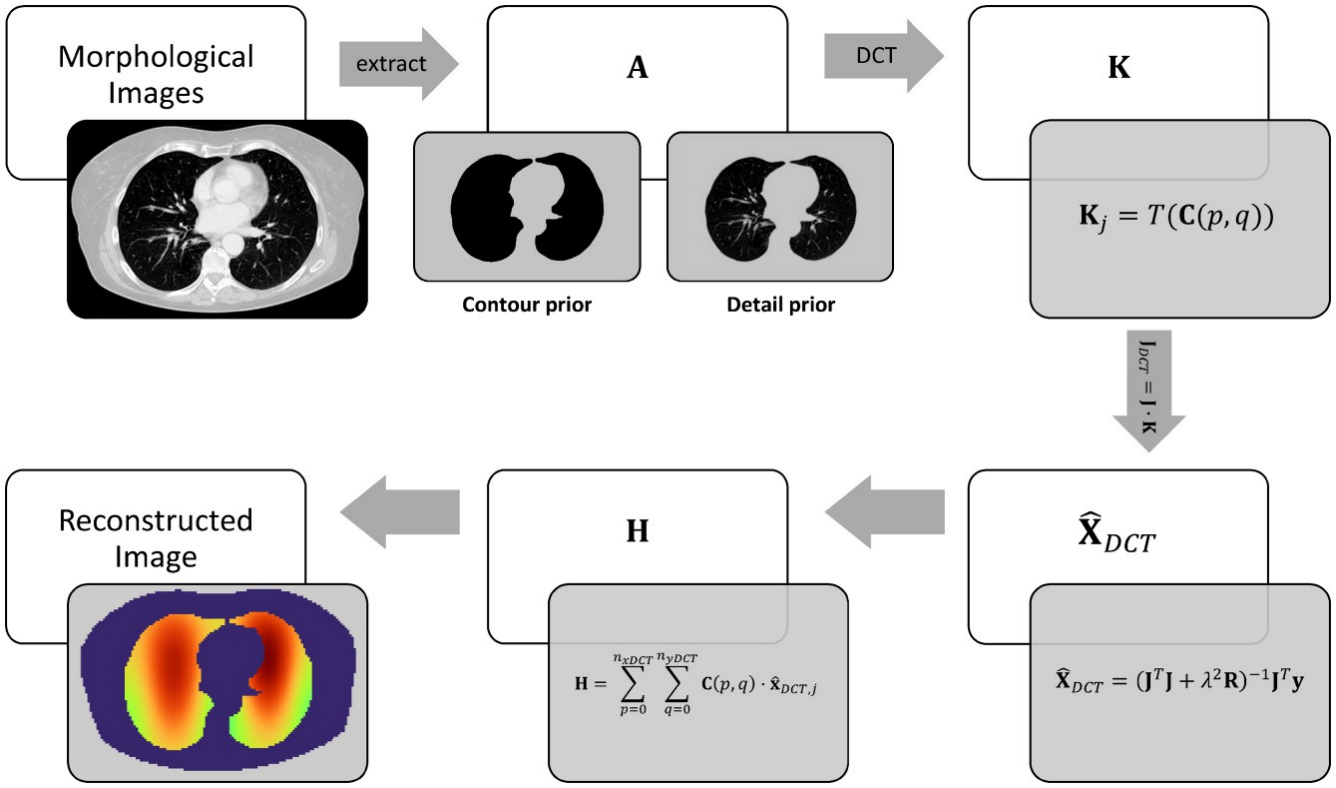
Procedure of the DCT-based EIT algorithm.

The change of DCT coefficients ${\hat{\text{x}}}_{\text{DCT}}$, which is the result of the reconstruction is used for the restoration of an EIT image **H**. With the matrix **C**(*p*, *q*) multiplied by the corresponding reconstructed ${\hat{\text{x}}}_{DCT,j}$ and summed element by element, an image **H** can be recovered. The image **H** has the same resolution as the original CT image (**A**) with *M* rows and *N* columns.

$$\begin{array}{c} \text{H}=\sum_{p=0}^{n_{xDCT}}\sum_{q=0}^{n_{yDCT}}\text{C}\left(p,q\right)\cdot {\hat{\text{x}}}_{DCT,j} \end{array}$$ (12)

where the *j* of the ${\hat{\text{x}}}_{DCT,j}$ is a function of the DCT frequencies as *j*(*p*, *q*) in the Eq 10. The restored image **H** consists of the prior structural information that was derived from the original CT image **A**. The entire procedure of the DCT-based EIT algorithm is depicted in Fig 3.

### Evaluation of the DCT-based approach

In the simulation assessment, we used two sets of forward models to simulate different lung conditions. Boundary voltages were generated, and then served as measurements in the EIT reconstruction process. The simulation ground truth is the best possible source of prior that can be used in the reconstruction. A contour prior and a detail prior were derived from simulation ground truth. With these two structural priors, the reconstructions from the DCT-based approach were compared with the reconstructions from both one-step Gauss-Newton solver and GREIT.

In the retrospective data analysis, six patient datasets were used (details in Table 1), including a long-term seven-day monitoring. CT images from the patients are used to generate detail priors for the DCT-based approach. The reconstructions from the DCT-based approach were compared with the GREIT reconstructions. The CT derived thorax shape was used in both algorithms.

**Table 1:** Patient characteristics.

Patient	Symptoms	Type of Breathing	EIT Monitoring Device
A	Pneumonia	10-25 cmH_2_O PEEP step	PulmoVista500
B	Pneumonia	10-25 cmH_2_O PEEP step	PulmoVista500
C	Pneumonia	0-15 cmH_2_O PEEP step	Goe-MF II
D	COPD	Spontaneous tidal breathing	PulmoVista500
E	COPD, Lung emphysema	Spontaneous tidal breathing	PulmoVista500
F	Pneumonia	0-15 cmH_2_O PEEP step	Goe-MF II

#### Simulation data

**Fig 4:**
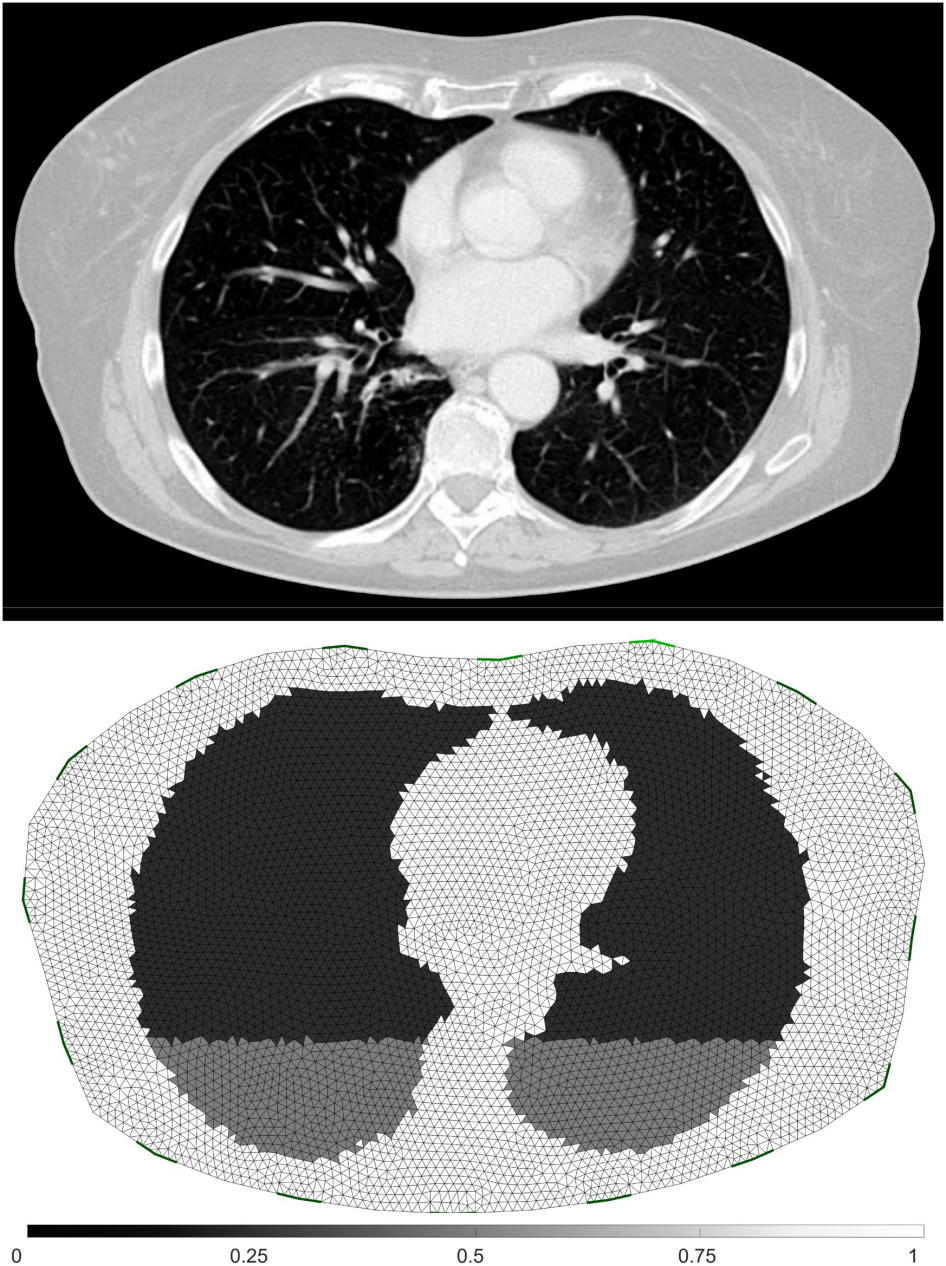
First row: The original CT image. Second row: The derived FEM model with 25% atelectasis area at the dorsal part.

To generate the simulated boundary voltages required for the evaluation of different EIT reconstruction methods, we used Matlab R2019b (Mathworks, Natick, MA) and the EIDORS toolbox [58], in which NETGEN [9, 59] was adopted to generate the finite element meshes (FEM). The thorax and lung contour in the simulated two-dimensional model with 122,306 generated FEM elements were derived from the CT image. Both the CT image and the derived FEM model are depicted in Fig 4. We implemented the adjacent stimulation pattern and adjacent voltage measurement pattern, which is often found in commercial EIT equipment. Initially, the conductivity of the elements within the lung area were set to *σ* = 0.5, and the rest of the elements are set to *σ* = 1. The boundary voltages corresponding to this initial setting were stored as **V**^*baseline*^, which was used as a reference frame in the image reconstruction. After the change of simulation settings, boundary voltage changes **V** were generated. Normalized voltage changes $\text{y}=\frac{\text{V}-{\text{V}}^{baseline}}{{\text{V}}^{baseline}}$ were used for subsequent reconstruction process. To mimic a more realistic measurement situation, 25% of Gaussian noise scaled to the standard deviation of **v**-**v**_*baseline*_ was superimposed to the boundary voltage measurements **v**.

**Fig 5:**
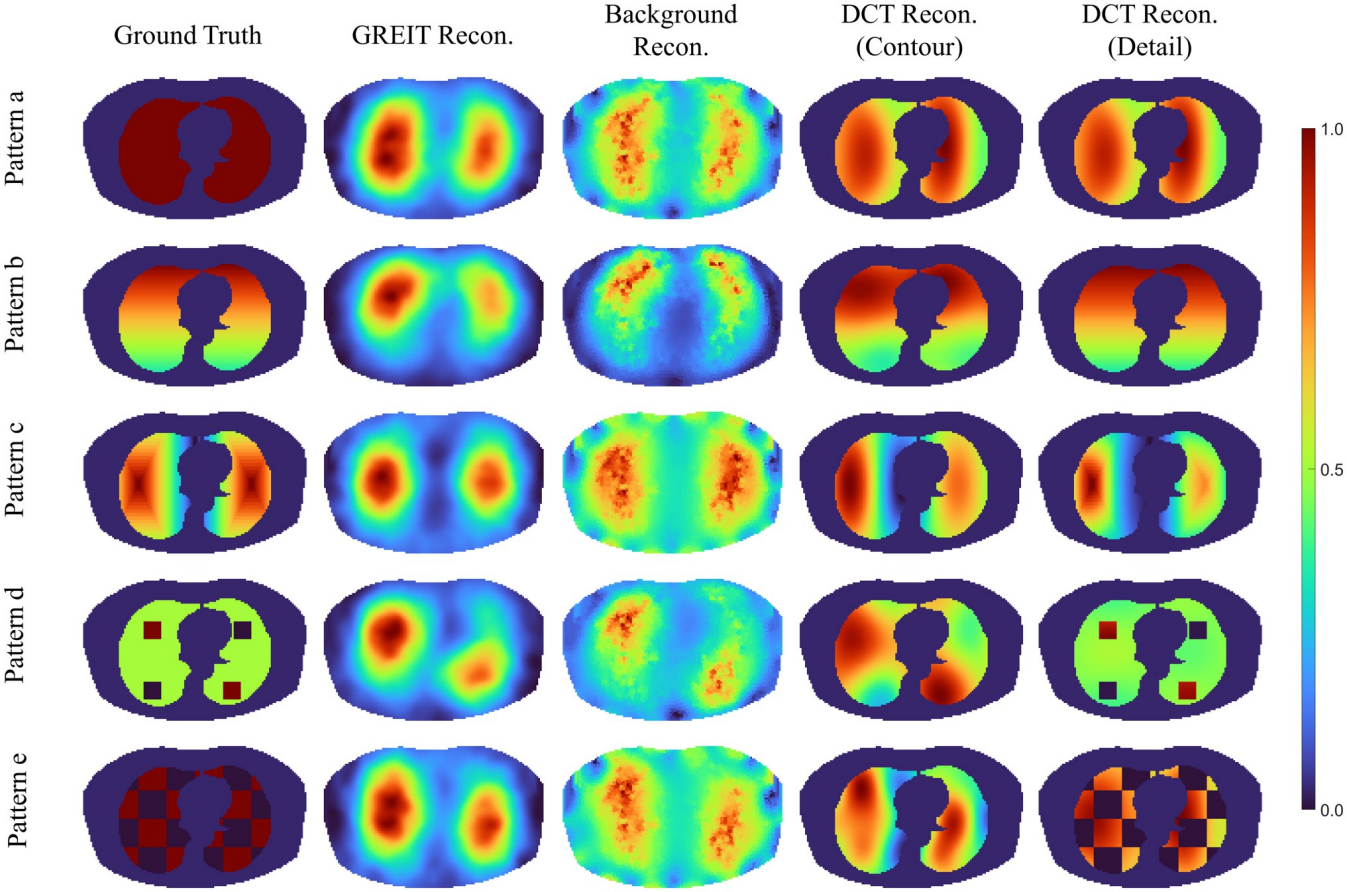
First column: Patterns of the conductivity distribution (normalized) implemented in the simulation for the measurement of the boundary voltage. The second to fifth column: Reconstructions of different algorithms, GREIT, background reconstruction with one step Gauss-Newton solver, DCT-based EIT approach with contour prior and DCT-based EIT approach with detail prior, respectively. All pixel values in images are normalized.

In the first part of simulation validation, five different and rather artificial patterns of conductivity distributions within the lung area were implemented (Fig 5, first column, shown as normalized pixel value) to generate the required “measurement” data for the reconstruction process:

a Homogeneous conductivity distribution of the complete lung area with *σ* = 0.25. With this uniform distribution all artefacts introduced by the reconstruction method can be identified;
b Gradient decreasing conductivity distribution with *σ* = 1 at ventral lung area. This pattern is similar to gravitational gradient (hydrostatic pressure gradient) induced lung properties;
c Gradient decreasing conductivity distribution with *σ* = 1 in the centre of lung;
d Four high contrast areas in the ventral and dorsal lung with *σ*_1_ = 0 and *σ*_2_ = 1, other parts of the lung remain unchanged. High contrast areas reveal the ability of a reconstruction method (e.g. its regularization) to reconstruct rapid changes in conductivity;
e Chess-board pattern of conductivity distribution with *σ*_1_ = 0 and *σ*_2_ = 1. A rather artificial pattern, which provides information about the resolution that is supported by the reconstruction method.

All the simulated conductivity distributions within a lung area are special cases in a condition to evaluate the algorithm in a clearly defined situation. It does not intend to mimic exact lung physiology or pathology. The conductivity is given in arbitrary units (AU).

The second part of simulation involves a lung model with different states of atelectasis, from 0% to 50%, in the dorsal part of the lung. This simulation shed some light on more realistic lung conditions. The same forward model derived from a patient dataset used in the first part of the simulation is used. One example with 25% atelectasis on the dorsal part is depicted in Fig 4. The dark grey dorsal part of the lung indicates the atelectatic portion.

For the DCT-based reconstruction process, EIT measurement **y** and structural priors from morphological images, e.g., CT, will be used. The setup of the simulation is to create an ideal situation: the real status of the lungs (the simulation ground truth) is known. Thus, the simulation ground truth is an ideal structural prior. The ground truth derived lung shape was used as a contour prior, and the ground truth derived weights were used as a detail prior. The simulation setting tries to describe the benefits of an accurate structural prior to the EIT reconstruction. Another FEM model with same thorax shape but different and coarse FEM meshes is used in order to avoid the ‘inverse crime’ [60].

To compare the difference between the reconstructed image and the ground truth, we calcualted the pixel wise *ℓ*_2_-norm of the image differences:

$$\begin{array}{c} \ell_{2}\;norm\;of\;image\;difference=\sqrt{\sum_{i=1}^{M}\sum_{j=1}^{N}{\left(H_{ij}^{Recon}-H_{ij}^{GT}\right)}^{2}} \end{array}$$ (13)

where the **H**^*Recon*^ is the EIT reconstruction, **H**^*GT*^ represents the simulation ground truth, *ij* is the index of each pixel. A lung mask **P** is used in the DCT-based algorithms to constrain the reconstruction within the lung area. To allow for a fair comparison in addition to the results from pure GREIT and one-step Gauss-Newton solver, reconstructed images with same lung mask **P** applied to both methods are calculated. Furthermore, EIT reconstruction shows the pixel value in arbitrary unit (AU), and different EIT algorithms yield different pixel value. To achieve comparable results, the reconstructions generated by the different algorithms were normalized to pixel values between [0, 1].

#### Patient data

Retrospective clinical datasets obtained from six patients (2 females, 4 males, 70yrs±5) were included (details in Table 1). The study was approved by the Human Investigation Review Board University of Szeged (approval number 67/2020-SZTE). Written informed consent was obtained from the patients or their legal representatives, the methods were carried out in accordance with the approved guidelines and regulations. Four patients were deep sedated, intubated and ventilated with a PEEP step maneuver performed, the other two patients were spontaneously breathing. A survey of patient characteristics is found in Table 1. Patient A was monitored for seven days, which allowed day by day comparison. All datasets are recorded either by the Dräger PulmoVista 500 device (Dräger, Lübeck, Germany) or by the Goe-MF II device (Viasys Healthcare, Höchberg, Germany). CT data of each patient were obtained at the first day of admission into the hospital. The CT image on the plane where the EIT belt was attached were used to determine the thorax contour for FEM. The details of the lung from the CT were used to generate the detail prior.

For the day-by-day evaluation using the data from patient A, reconstructions from the DCT-based approach were compared with that of GREIT. In addition, global inhomogeneity (GI) indices [61] calculated from DCT-based approach reconstructions were compared with those from GREIT. GI index is calculated from an EIT tidal image using Eq 14:

$$\begin{array}{c} GI=\frac{\sum_{ij}\|H_{ij}^{lung}-median\left(H^{lung}\right)\|}{\sum_{ij}H_{ij}^{lung}} \end{array}$$ (14)

where **H**^*lung*^ is the lung region of an EIT tidal image, *ij* is the index of a pixel within the lung region. In our research, in order to have comparable results, the lung region, which is derived from the CT, was kept same in GI calculation.

## Results

### Simulation data

Different reconstruction algorithms, GREIT, one-step Gauss-Newton solver and the DCT-based approach, were applied to the five introduced conductivity distributions. The results are depicted in Fig 5. As different algorithms have different EIT pixel ranges in the reconstruction, all images were normalized between [0, 1] in Fig 5. The first column depicts the conductivity distribution in the simulation model, which served as a ground truth for simulation and evaluation. The images reconstructed with GREIT or background reconstruction with one-step Gauss-Newton solver are shown in the second and third column, respectively. The DCT-based approach was implemented with two different priors, in the fourth column are the results with a contour prior and the fifth column depicts the reconstruction based on the detail prior derived from simulation ground truth. Reconstructions are displayed using the same colour range.

**Fig 6:**
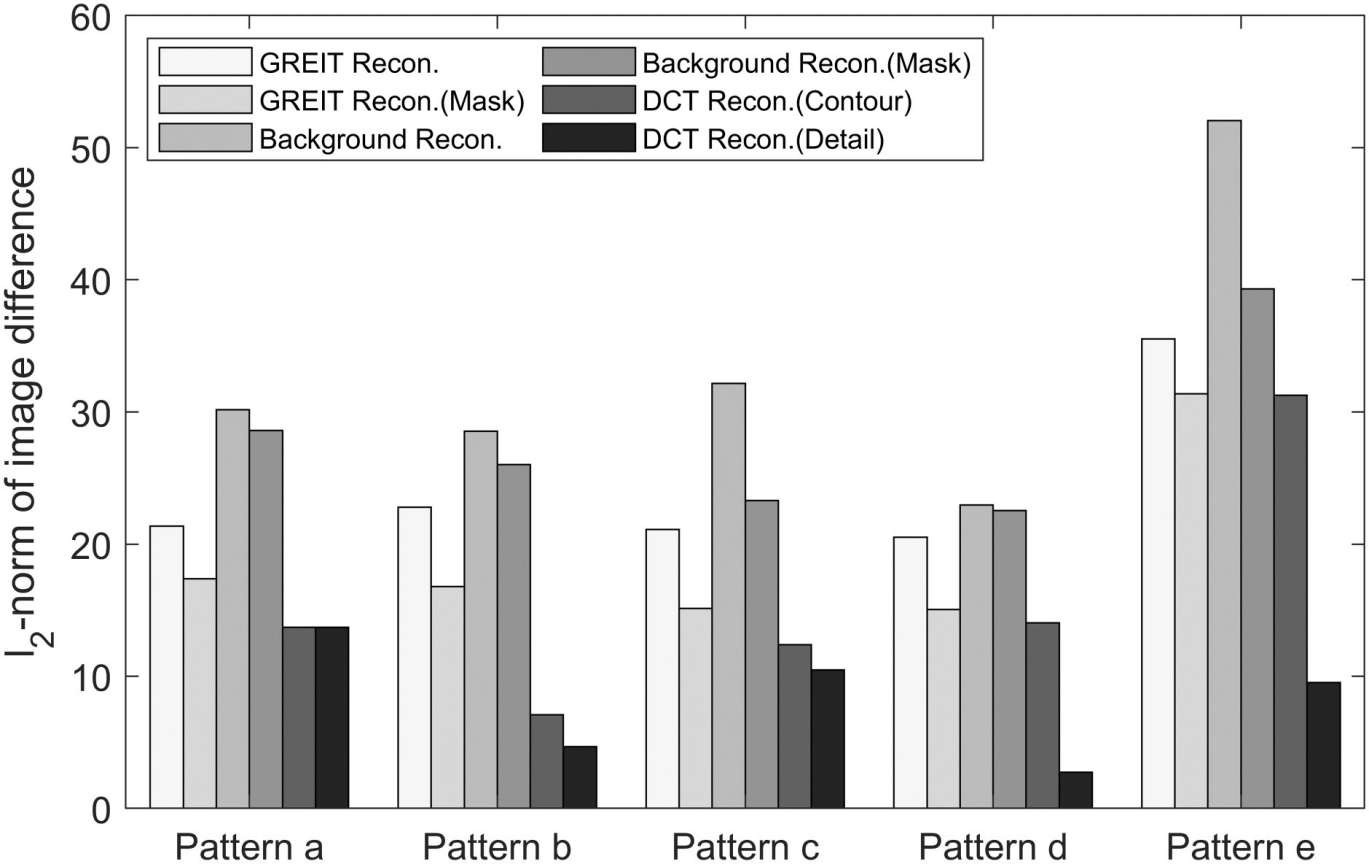
The *ℓ*_2_-norm of image difference between the respective reconstruction result and the ground truth.

From the results, it is obvious that reconstructed images are influenced by different algorithms, though based on the same boundary measurement **y**. The DCT based reconstructions do not show artefacts outside of the lung region and show clearer results. For a quantification of the comparison, the *ℓ*_2_-norm of image differences between the ground truth and the reconstructed images were calculated and are shown in Fig 6. Large, but systematic differences with respect to the simulation model and to the methods are obtained. It demonstrates the relation of image quality and information content implemented into the reconstruction priors. The DCT-based EIT algorithm with the detail prior shows the most accurate reconstruction results. But even a quite unspecific lung contour prior with the DCT-approach still demonstrates significant improvements over the other included methods.

**Fig 7:**
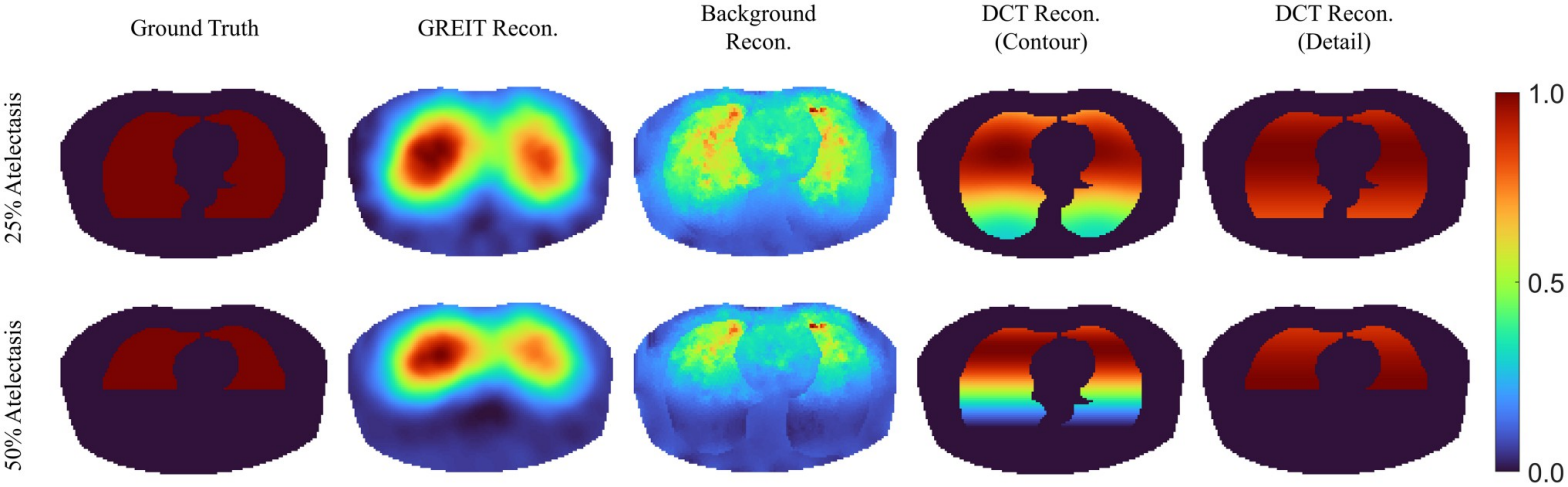
Left column: Example atelectasis scale used as ground truth in the simulation. The second to fourth column: Reconstruction images of different algorithms, GREIT, background reconstruction with one step Gauss-Newton solver, DCT-based EIT approach with contour prior and DCT-based EIT approach with detail prior, respectively. All pixel values in images are normalized.

Fig 7 demonstrates the reconstructed results from different algorithms in the simulation of dorsal atelectasis, where two levels (25% and 50%) of atelectasis were selected. The DCT-based approach produces very clear results, but it still overestimates the conductivity in the atelectatic area if only the contour prior is applied. With detail prior, the reconstruction is inhibited within the atelectasis area. The GREIT reconstruction is quite accurate as well showing almost no change within the atelectatic area.

**Fig 8:**
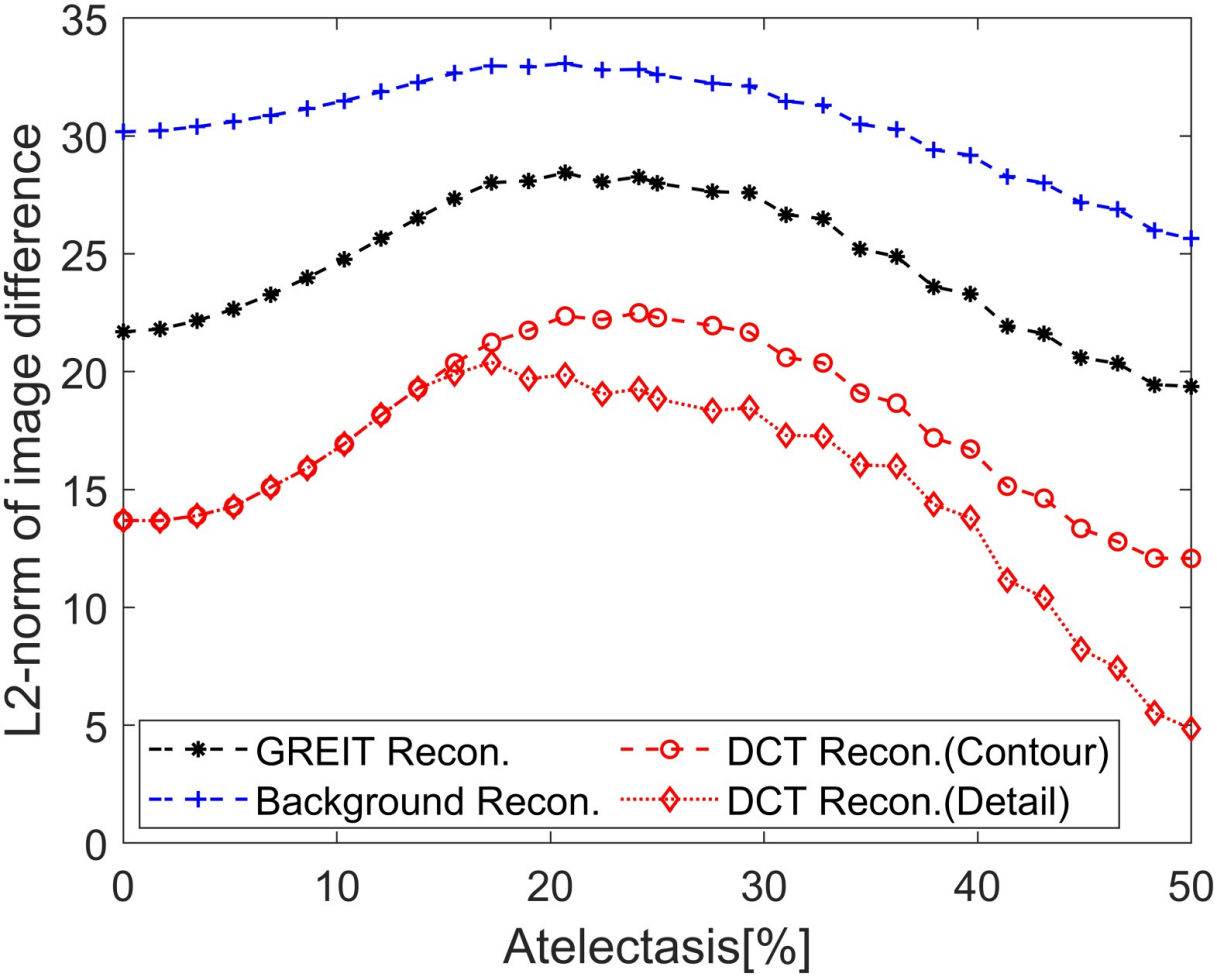
The *ℓ*_2_-norm of the image difference between the reconstructed image and the ground truth for a certain scale of atelectasis. Black asterisk: GREIT reconstruction; blue cross: reconstruction with background prior; red circle: DCT-based approach with contour prior, red diamond: DCT-based approach with detail prior.

The *ℓ*_2_-norm of the image difference between the reconstructed image to the ground truth were illustrated in Fig 8. The result confirms the observation in the first part of the simulation: the DCT-based EIT algorithm with detail prior shows the most accurate reconstruction results. However, before the atelectasis level increased to 15%, the *ℓ*_2_-norm of the image differences of the DCT-based EIT algorithm with either detail prior or contour prior appeared to be almost identical.

### Patient data

**Fig 9:**
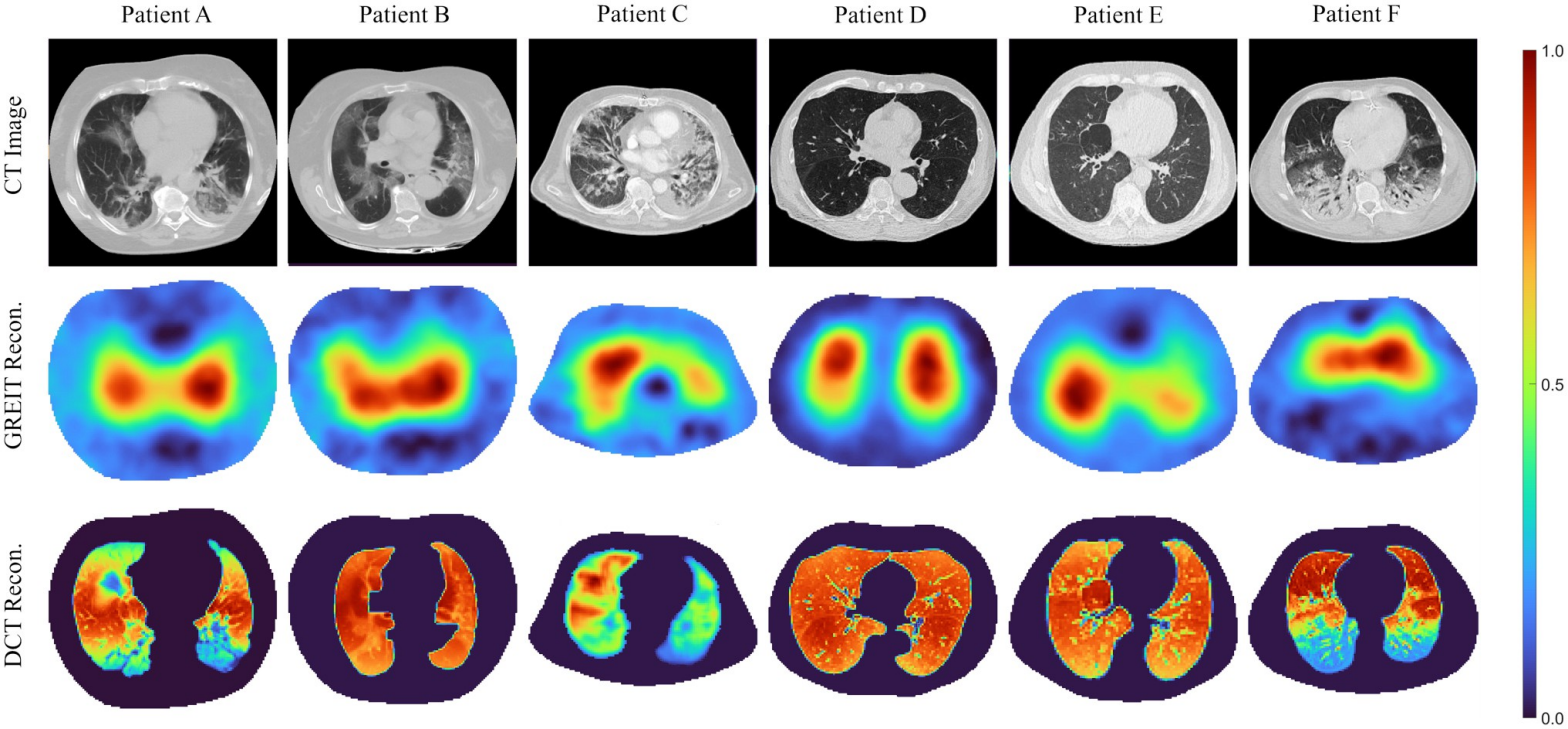
First row: CT images of the patient, which are used for the segmentation of the lung and thorax shape for EIT reconstruction. Second row: GREIT reconstructions of conductivity change. Third row: Reconstructions of conductivity change using the DCT-based approach with detail prior derived from the related CT image.

From six patients with a recent individual CT available, one EIT frame from each patient is demonstrated in Fig 9. The corresponding CT images are shown in the first row in Fig 9. The GREIT reconstructions are shown in the second row, while the reconstruction of the DCT-based approach with detail prior are depicted in the third row.

**Fig 10:**
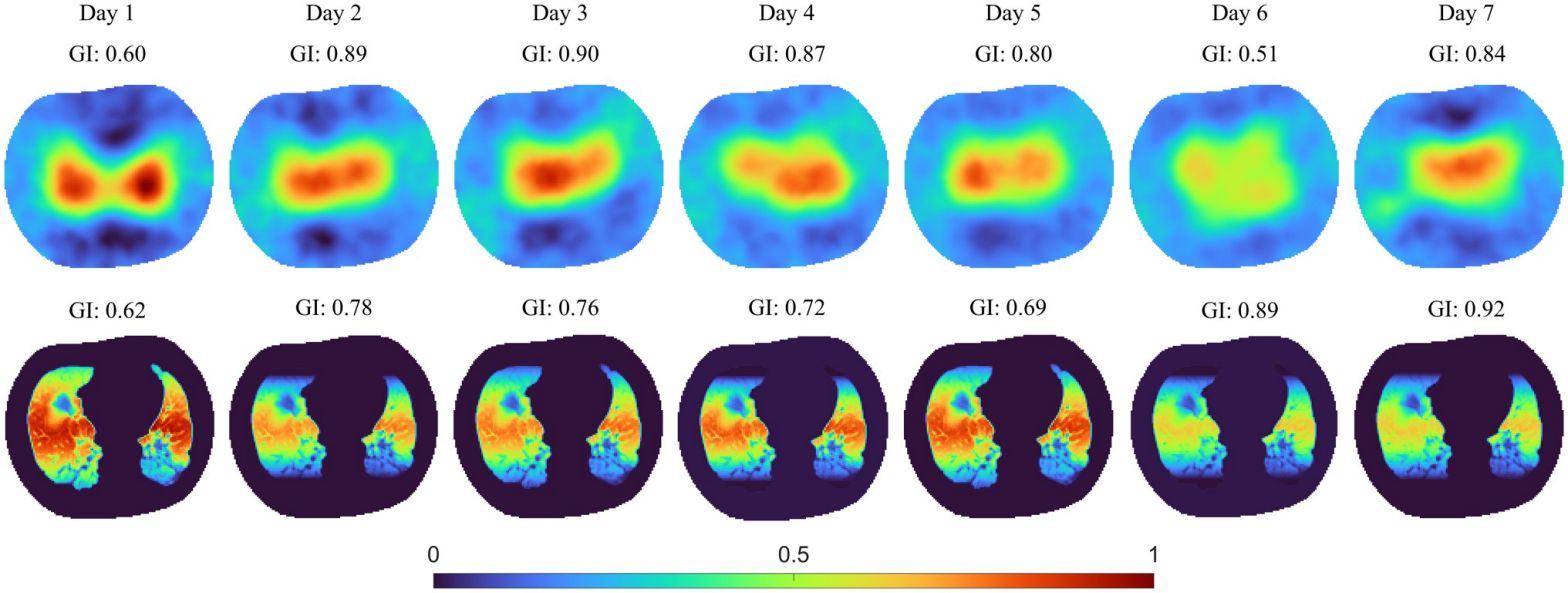
A seven-day monitor procedure of the patient A at the PEEP stage of 25 cm H_2_0. GI indices were calculated and provided above each image. First row: GREIT reconstructions of conductivity change; second row: Reconstructions of conductivity change using the DCT-based EIT approach with detail information prior derived from the related CT image.

Obviously, the true conductivity distribution in each patient is unknown, which prohibits the simple *ℓ*_2_-norm of the image difference analysis in simulation studies. Nevertheless, plausibility of the results according to the medical documentation can be judged. In patient A, atelectasis at the left dorsal lung part was observed, which is in accordance with the negligible conductivity variation in the left dorsal lung area found in the DCT-based reconstruction. Similarly in patient C who suffered from atelectasis in the left lung area, little variation is observed in the DCT-based result in the respective area. Patient F had atelectasis at both dorsal lung parts, which is confirmed by both the CT image and the DCT-based results. Patient B was diagnosed with pneumonia and inflammation at both dorsal lung areas but was reported to have a quite good reaction to a PEEP manoeuvre. Inspection of the DCT-based EIT image also suggests that ventilation of dorsal parts is possible but with less air than in other parts of the lungs. A homogeneous ventilation distribution is observed in patient D who showed no abnormalities in the CT. Patient E was claimed to have pulmonary emphysema at both ventral parts, so less ventilation at the ventral parts can be seen from the DCT-based result. The GREIT results show a good agreement, i.e., indicating a region where the ventilation occurs, but without morphological details for more interpretability. Fig 10 illustrates a consistent seven-day monitoring on patient A with the reconstruction results taken from the same PEEP level and depicted in the same colour scale for comparison. GI index for each PEEP level is shown above the reconstruction. Patient A was reported to undergo an exacerbation on the third day and the sixth day [62], which is easily recognised in both algorithm results depicted in Fig 10 as less ventilation is found in the reconstructions. However, the ventilation variation in the GREIT results show some deviations on day 3 to 5. The GI indices of both GREIT and the DCT-based approach support the same changing trend.

## Discussion

Contour prior and detail prior were integrated into the DCT-based EIT reconstruction, and the results were compared to the well-known GREIT algorithm or one-step Newton-Gauss solver. It was demonstrated that there is a straightforward way to incorporate simple or complex structural priors into the EIT reconstruction. The results clearly indicate that a correct structural prior, e.g., a valid detail prior, leads to superior reconstruction result in the DCT-based approach. Especially the DCT-based approach provides a natural way to fuse structural prior into functional EIT reconstruction. The results from the DCT-based approach show less error (*ℓ*_2_-norm difference to simulation ground truth) when compared to the one-step Newton-Gauss solver (basic FEM element clustering) or to GREIT. This finding confirms reports about the beneficial effect of good structural priors, e.g., a subject specific anatomically accurate forward model produced better reconstruction than the uniform background [55].

Achieving a more interpretable and personalised EIT reconstruction is a significant advance for the routine EIT application in clinical settings. Different approaches that use patient related structural priors to improve the reconstruction results are also reported by several other research groups. Nakanishi et al. established an anatomical atlas consisting of probability distributions of tissue conductance obtained from measurements in multiple patients [63]. It is used as a general prior in a Bayesian reconstruction approach, which is much less computationally efficient and not personalized. Other work confirmed that the homogeneous background assumption of most commercially available reconstruction methods may lead to clinically relevant errors in the reconstruction [55]. This observation indicates a need of effective methods to integrate individual prior information. In Zhang et al., machine learning techniques are used to efficiently incorporate structural elements into the reconstruction process [64]. This technique might be a candidate to evaluate the validity of structural priors in the reconstruction.

GREIT, whose Jacobian matrix is optimized to figures of merit, agreed by experts in the field, has already achieved very good results. Reconstructed images show reduced position errors and small ringing artefacts, but still require a target training procedure on a 3D-FEM model and were always inferior to the DCT-based approach. The DCT-based EIT algorithm has shown its ability to reconstruct a more accurate conductivity distribution even in extreme situations with sharp edges or ridges. Since the DCT-based EIT reconstruction is using the simple but efficient one-step Gauss-Newton solver with a direct mapping to restrain the reconstruction into a morphological lung area, it comes merely without extra computational cost. On the contrary with careful selection of the DCT dimensions, a fast 3D-approach can be implemented in further research due to the reduced degrees of freedom in the reconstruction [43]. In addition, the GREIT reconstruction showed an imbalance conductivity distribution between the left lung and the right lung, e.g., pattern b in Fig 5, regardless the similar conductivity distribution in either lung area, but the results from DCT approaches witnessed the similar distribution at either side.

Furthermore, the DCT-based approach introduces a personalised structural prior, e.g., CT derived lung contour, into the EIT reconstruction process. The morphological structure of the lungs is well preserved both in the reconstructions from simulations and in retrospective clinical datasets. In prospective clinical settings, the regional information in terms of functional status of the lungs can thus be directly correlated with the lung structure, providing a comprehensive insight of the pathophysiology of the lungs. In addition, morphological images, e.g., CT, should be available for patients that require EIT monitoring, as EIT monitoring is generally used on patients with mechanical ventilation support. The CT structure is embedded into the reconstruction, where the FEM is constructed from CT contour information and via matrix **C** and mapping *T*, i.e., the reconstructed image is overlaid with the CT image without any additional registration process.

The beneficial effect of correct structural priors could be shown in the simulated cases, but there is a risk associated with priors if they are old or wrong. Misleading results can be produced from wrong or partially incorrect prior. In the simulation-based evaluation part, the DCT-based EIT algorithm will generate appealing results when a detail prior from ground truth is specified. For example, prior information in the rather artificial Pattern e indicates the conductivity distribution is in form of a chessboard, where the detail prior indicates no variation should be expected in some locations. This prior will result in an inaccurate reconstruction if the simulation setting changes. Or in other words, this static prior will be wrong when the conductivity distribution deviates from chessboard like structure.

In most clinical settings, CT or MRI images are the source to form the basis for the patient personalised structural prior, but they will not be up to date when EIT might be applied. Usually, medical imaging is obtained when the patient is admitted to the hospital. Those images do not reflect the course of the disease or the developing status of the patients’ organs at the time when the EIT is taken. Nevertheless, they can provide a useful bias that is adjusted by the measured data during reconstruction. In addition, with the help of physiological modelling and clinical data, morphological information could be updated over time by predicting potential changes based on pathophysiology and available patient measurements. If this is a feasible approach remains to be shown in the futures. For example, during the seven-day monitoring of patient A (cf. Fig 10), the left dorsal part of the lung showed little change as this area was an atelectasis area in the CT. However, we cannot prove the atelectasis area remains the same as no further CT data are available in the following days. However, the DCT-based EIT reconstruction still indicates the status of the patient as the deteriorating trend is clear and complies with the medical record.

It is worthwhile to mention that the reconstructions of the two different algorithms, GREIT and the DCT-based EIT algorithm, reveal different information in Fig 10. In the GREIT reconstructions, from day 3 to day 4, there exists a ventilation distribution shift from the right lung to left lung, and then the ventilation distribution shifted back to the right lung on day 5. This distribution shift is not observed in the DCT-based EIT results. However, except for the shift, the changes of the ventilation related conductivity variations are still obvious and correlated between the two methods. The GI indices also support the same changing trend in both two methods. It is not clear which algorithm is correct in this case. The shifting in GREIT images might be a result from positioning the patient from one side to the other (but which is not documented in the patient record), or the unlikely effect that virus inflammation is switching back and forth between lung lobes or may be due to numerical effects in the regularization process. To clarify the clinical situation would require expensive and potentially harming imaging methods, e.g., single-photon emission CT, which was not available at the bedside.

There are multiple limitations of the contribution. The clinical retrospective data only includes six patients, and simulations are restricted to a limited number of model cases. Still, they demonstrate nicely the principles of structural priors. More patients with different type of lung disease should be included to identify more cases with contradicting results between reconstruction algorithms. For the long term EIT monitored patient A, the EIT measurement was conducted during the high peak of COVID-19 pandemic, we cannot assure that the EIT belt, as well as electrodes, was applied on the same position of the patient during the seven-day period. There is a possibility that EIT measurement could be corrupted on some day. However, the decreasing trend of recruitment and the exacerbation on the third day and the sixth day, which is shown in Fig 10, were confirmed with the doctor [62]. More clinical trials in terms of longer and consecutive monitoring should also be considered into our future research to verify the ability of this novel algorithm in detecting the course of disease, and especially when a structural prior is outdated. Furthermore, this comparison only includes two other algorithms with roughly the thorax and lung contour specified in the inverse FEM model. The comparison merely involves the visual and pixel wise image analysis in simulations. Further investigations will consider some clinical and EIT indices to compare their stability in clinical data over different implementations with prior driven EIT reconstruction algorithms. It is worth noting that the simulation experiments were carried out using a two-dimensional FEM model, but EIT is sensitive to the area cranial and caudal of the plane of electrodes in reality. A three-dimensional FEM model can provide more realistic boundary measurement. In addition, phantom experiments will be very helpful when validating novel algorithms and bringing them into clinical use. Carrying out phantom experiments is necessary for further development of this algorithm. Another concern raised is the intensive computational requirement of the proposed DCT approach, which may affect real-time analysis of EIT images. However, our proposed DCT approach does not require recalculation of the DCT subset or the structural prior unless there is a significant change in patient status. Additionally, specialized algorithms, such as presented in [65], are taken into the consideration to reduce the computational intensity of DCT significantly. This will allow it to be performed in real-time on standard clinical hardware.

In this study, since the access of free parameters is largely reduced by the DCT-based approach, regularization is not so decisive. Only structural priors were included but the rich work on regularization has been neglected, for example, total variation, which is a form of prior knowledge that can be combined with the structural priors that worth further investigation [51, 53, 61, 66]. However, the choice of regularization is not exclusive. All general regularization methods covered by Eq 5 can be combined with the presented method to incorporate structural priors. There is no need to limit the regularization matrix to the Tikhonov prior that was used in the presented comparisons.

With the structural priors, either contour or detail, in the DCT approach, the interpretation of the EIT derived functional images appears much easier for clinical staff but may carry the risk to overestimate EIT resolution and accuracy. However, it is worth noting that the morphological pattern in DCT-based results comes from CT-derived structural prior, rather than the EIT measurement. The validity of these structural priors should be checked carefully. Further research must be carried out to develop a plausibility test for the validity of the structural prior. Priors from morphological images might lose validity because a clinical setting is a dynamical system, e.g., showing patient disease status changes or treatment modifications like prone vs. supine positioning. The outdated prior information might induce a risk in terms of misleading interpretation of the results and might eventually compromise the diagnosis. A strategy is required first to carefully select the appropriate prior and second to detect, if the prior is outdated and misleading. Chen et al. have proposed a method to quantify the error introduced by a structural prior, which can evolve into an indicator of a false prior [67].

EIT evaluation in clinical setting should reveal useful information for decision making. Interpretability and easy access to the information provided by the DCT-based approach is helpful in this respect. For example, detection of overdistention can prevent the patients from lung damage cause by inappropriately high PEEP settings at the ventilator during the treatment of ARDS patients [68, 69]. The reconstructions produced by the DCT-based EIT algorithm demonstrate the potential to visually detect atelectatic regions, with the individualized lung morphology superimposed. It is worth noting that incorporating structural priors from other morphological images into EIT reconstruction is not limited to the DCT-based algorithm alone, and has the potential to be applied to other algorithms. Further exploration of this potential can lead to improved interpretability of EIT images of other algorithms, which may have practical implications in personalized protective ventilation treatment.

## Conclusion

The DCT-based EIT algorithm has shown the ability to reconstruct the regional inhomogeneous distribution of the ventilation correlated with the morphological information obtained from patient CT data. In addition, the reconstruction result can imply a developing course of the disease. Anomalies of ventilation distribution shown in the results from the DCT-based approach is correlated with the morphological information, which leads to an improved interpretation. However, the priors in the DCT-based approach may vice versa lead to inaccurate results if the pathological status changes with respect to the time of CT recording. Structural priors implemented in the algorithm can eventually become wrong or outdated with the course of disease and the pathophysiological development of the patient status, e.g., dependent atelectasis might be increased because of an interstitial lung edema [70]. Instead of an interpretable reconstruction, the prior implemented algorithm might yield a misleading result compromising the accuracy of the clinical decisions. Further research should be conducted in terms of the detect of outdated priors to ensure the accuracy of prior information, which might facilitate the diagnostic and monitoring procedure with EIT reconstruction in a clinical setting.
